# Single-cell mRNA profiling reveals transcriptional heterogeneity among pancreatic circulating tumour cells

**DOI:** 10.1186/s12885-017-3385-3

**Published:** 2017-05-31

**Authors:** Morten Lapin, Kjersti Tjensvoll, Satu Oltedal, Milind Javle, Rune Smaaland, Bjørnar Gilje, Oddmund Nordgård

**Affiliations:** 1 Department of Haematology and Oncology, Stavanger University Hospital, N–4068 Stavanger, Norway; 2 Laboratory for Molecular Biology, Stavanger University Hospital, N–4068 Stavanger, Norway; 3 Department of Mathematics and Natural Sciences, University of Stavanger, N–4036 Stavanger, Norway; 4 Department of Gastrointestinal (GI) Medical Oncology, Division of Cancer Medicine, MD Anderson Cancer Center, The University of Texas, Houston, TX USA

**Keywords:** Circulating tumour cell, CTC, Single-cell isolation, mRNA, RT-qPCR, Pancreatic cancer

## Abstract

**Background:**

Single-cell mRNA profiling of circulating tumour cells may contribute to a better understanding of the biology of these cells and their role in the metastatic process. In addition, such analyses may reveal new knowledge about the mechanisms underlying chemotherapy resistance and tumour progression in patients with cancer.

**Methods:**

Single circulating tumour cells were isolated from patients with locally advanced or metastatic pancreatic cancer with immuno-magnetic depletion and immuno-fluorescence microscopy. mRNA expression was analysed with single-cell multiplex RT-qPCR. Hierarchical clustering and principal component analysis were performed to identify expression patterns.

**Results:**

Circulating tumour cells were detected in 33 of 56 (59%) examined blood samples. Single-cell mRNA profiling of intact isolated circulating tumour cells revealed both epithelial-like and mesenchymal-like subpopulations, which were distinct from leucocytes. The profiled circulating tumour cells also expressed elevated levels of stem cell markers, and the extracellular matrix protein, *SPARC*. The expression of *SPARC* might correspond to an epithelial-mesenchymal transition in pancreatic circulating tumour cells.

**Conclusion:**

The analysis of single pancreatic circulating tumour cells identified distinct subpopulations and revealed elevated expression of transcripts relevant to the dissemination of circulating tumour cells to distant organ sites.

**Electronic supplementary material:**

The online version of this article (doi:10.1186/s12885-017-3385-3) contains supplementary material, which is available to authorized users.

## Background

Pancreatic cancer is one of few cancer types for which survival has not substantially changed over the last few decades. In Norway, the 5-year survival remains a meagre 7% [1], despite improvements in median survival demonstrated recently with novel multidrug treatments [2–4]. The poor survival associated with pancreatic cancer can be explained by the late clinical presentation, the aggressive disease trajectory, and the generally poor response to chemotherapy [5]. In addition, tumour biopsies are often inadequate for molecular testing, and there are few validated blood-based diagnostic or predictive biomarkers. Thus, there is a need for new biological markers that can improve diagnostics by identifying localized disease and that can predict tumour progression and resistance to systemic therapy. Circulating tumour cells (CTCs) have potential as a biomarker, because they represent a “snapshot” of the total tumour burden, and they provide information about the tumour of origin. Additionally, because they are associated with the migration of cancer to distant sites, they may also indicate the underlying biology of the metastatic process.

To date, the small number of studies on the clinical relevance of CTCs in pancreatic cancer have produced ambiguous results (reviewed in [6, 7]). In addition, they have been limited to analysing CTCs with only one or a few markers, which is insufficient to elucidate the complexity of CTC involvement in the metastatic process. Investigations into the mutational landscape of primary pancreatic carcinomas and metastases have shown that specific mutations are only present in a small subset of tumour cells, and that the mutational profile of metastases may be different from that of the primary tumour. Thus, heterogeneity exists among tumour cells and among tumour sites [8, 9]. Some of these cell subsets may also be clinically relevant, because they may harbour specific mutations associated with therapy resistance and disease progression. The heterogeneity among tumour cells is further expected to be apparent at both the transcriptional and translational levels. Thus, because the CTC population is a “snapshot” of the total tumour burden, its characterization in analyses at the single-cell level could provide valuable information. The CTC population is also affected by the epithelial-mesenchymal transition (EMT); a process which changes the phenotype and migratory properties of CTCs. Moreover, it has been suggested that the EMT is involved in the dissemination process [10]. Thus, single-cell analyses might reveal CTCs with different transcriptional and mutational profiles, and a characterization of these subtypes could identify CTC phenotypes that are involved in dissemination to distant organ sites. In a previous study, heterogeneous expression of RNA transcripts was demonstrated in CTCs with single-cell mRNA profiling in samples from a small cohort of patients with pancreatic cancer [11]. To our knowledge, no other study has described transcriptional heterogeneity among single pancreatic CTCs and its potential clinical relevance.

To characterize pancreatic CTCs molecularly at a single-cell level, we applied a multi-marker negative depletion strategy, known as MINDEC [12], to peripheral blood samples from patients with locally advanced or metastatic pancreatic cancer. With single-cell multiplex mRNA profiling, we demonstrated that CTCs from patients with pancreatic cancer comprised distinct, epithelial-like and mesenchymal-like subpopulations. In addition, the CTC population showed enriched expression of cancer stem cell (CSC) markers and the extracellular matrix (ECM) protein, *SPARC*.

## Methods

### Patient samples

Between March 2015 and January 2017, we included 21 patients with locally advanced (*n* = 2) or metastatic (*n* = 19) pancreatic cancer treated with chemotherapy (nab-paclitaxel plus gemcitabine or FOLFIRINOX) at Stavanger University Hospital. Peripheral venous blood samples were drawn at multiple time points, before (*n* = 8) and after (*n* = 48) the start of chemotherapy in 9-mL EDTA tubes, and processed within 2 h. The treatment response was defined with standard disease evaluations of images, based on the RECIST 1.1 criteria [13]. All patients and healthy controls provided written informed consent to participate in the study. The project was approved by the Regional Committee for Medical and Health Research Ethics (REK-Vest 2011/475).

### Cell line cultivation and spiking

Human pancreatic cancer cell lines, ASPC-1 and PANC1, and the human mesothelioma cell line, SDM103T2, (all from ECACC) were cultured according to manufacturer recommendations, except that the culture media was supplemented with 100 units/mL penicillin and 0.5 mg/mL streptomycin (Penicillin-streptomycin, Sigma-Aldrich). Cells were harvested by adding 0.25% trypsin/EDTA (Sigma-Aldrich) for 3–5 min at 37 °C. For spiking experiments, 1000 cells were spiked into 9 mL of blood from a healthy volunteer.

### CTC enrichment

All blood samples were processed with density gradient centrifugation using Lymphoprep™ density gradient medium (Axis Shield, Norway), according to manufacturer instructions. After centrifugation, mononuclear cells were resuspended in 1 mL isolation buffer (PBS supplemented with 2 mM EDTA and 0.1% BSA) and layered on top of 3 mL foetal bovine serum to remove residual platelets. Subsequently, the samples were centrifuged at 200×g for 15 min at room temperature, and platelets were removed with the supernatant. The samples were then resuspended in 100 μL isolation buffer and processed with the MINDEC strategy [12]. Briefly, each sample was labelled with biotinylated antibodies directed at CD45, CD16, CD19, CD163, and CD235a/GYPA. Then, streptavidin-coated super-paramagnetic beads (Depletion MyOne™ SA Dynabeads®, Life Technologies AS, Norway) were added, and magnetic force was applied to remove bead-bound leucocytes and any remaining erythrocytes. Unbound cells in the supernatant were collected for subsequent analyses.

### Immuno-fluorescence labelling

Enriched cells were resuspended in 100 μL cold staining buffer (PBS supplemented with 2 mM EDTA and 0.5% BSA), with 25 μL FcR blocking reagent (Miltenyi Biotech), 2 μL Hoechst 33,342 (Molecular Probes), and 2 μL of each of the labelled antibodies EpCAM-FITC (clone HEA-125, Miltenyi Biotech), MCAM-FITC (clone OJ79c, AbD Serotec®), and CD45-DyLight550 (clone T29/33, Leinco Technologies, Inc.). Then, cells were incubated in darkness for 20 min at room temperature. Stained samples were washed with staining buffer, and subsequently resuspended in 150 μL cold staining buffer.

### Single cell isolation

**Fig. 1:**
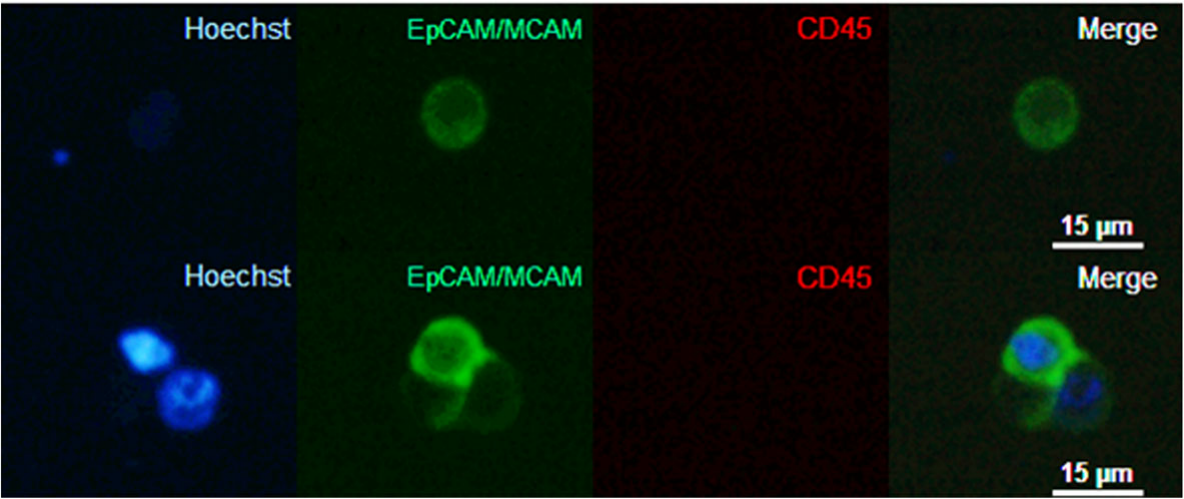
Thumbnail gallery of analysed CTCs. Representative fluorescence images of a CTC (*top row*) and a CTC cluster (*bottom row*) isolated from the peripheral blood of a patient with pancreatic cancer. (*Left column*) Hoechst stain (blue) identifies nuclei; (*second column*) EpCAM-MCAM (*green*) identifies membranes of CTCs; (*third column*) CD45 (*red*) identifies leucocytes; (*right column*) superimposed images confirms intact cells. Images were acquired with 20× magnification. To enhance visibility, we adjusted the brightness and contrast equally for all microscopic images, and these adjustments were applied to the entire image

The stained cell suspensions were transferred to a microscope slide coated with Silanization Solution I (Sigma) and imaged with an Olympus XI 81 inverted microscope (20× magnification, Olympus LUCPLFLN 20×). Exposure times were fixed at 86 ms for Hoechst 33,342, 1400 ms for EpCAM-MCAM, and 1600 ms for CD45. Each sample was manually inspected to identify tumour cell-line cells or possible CTCs. Cells with a visible nucleus, no beads attached, expression of EpCAM-MCAM, no expression of CD45, and with a round or ovoid shape were classified as cell-line cells or CTCs (Fig. 1) and subjected to single cell isolation. A threshold for EpCAM-MCAM positivity had been established previously by processing blood samples from healthy volunteers [12]. The selected cells were transferred into 8.9 μL lysis buffer (9 parts Lysis Enhancer and 1 part Lysis Solution, Invitrogen) on a hydrophobic microscope slide with a MMI CellEctor cell manipulator (Molecular Machines & Industries). Then, cells were transferred into PCR tubes by manual pipetting, and subsequently they were frozen at −80 °C until further analysis. Possible CTC clusters (≥2 CTCs) were also isolated and analysed as a single entity.

### Single-cell RT-qPCR

Reverse transcription and pre-amplification were performed with the CellsDirect™ One–Step qRT–PCR Kit (Invitrogen, Carlsbad, CA). Briefly, isolated single cells stored in lysis buffer were thawed on ice and lysed by incubation at 75 °C for 15 min. Subsequently, to each lysed cell, we added 5 μL DNAse I, Amplification Grade (1 U/μL) and 1.6 μL 10× DNase I buffer (both Invitrogen). The reactions were incubated at room temperature for 5 min. Next, 4 μL of 25 mM EDTA (Invitrogen) was added to each reaction, and the mixture was incubated at 70 °C for 10 min. Next, we added 1 μL SuperScript® III RT/Platinum® Taq Mix, 25 μL 2× Reaction mix (both Invitrogen), and 4.5 μL of 13 pooled 20× TaqMan assays (Applied Biosystems) at a 1:40 dilution. Single-cell mRNA was then reverse-transcribed to cDNA (50 °C for 15 min, 95 °C for 2 min), pre-amplified for 14 cycles (each cycle: 95 °C for 15 s, 60 °C for 4 min), and subsequently, diluted 1:5 in TE buffer.

Quantitative PCR was performed by mixing 2 μL of diluted pre-amplified cDNA with 9.25 μL nuclease-free H_2_O, 12.5 μL TaqMan Gene Expression Master Mix, and 1.25 μL 20× TaqMan assay. The thermocycling protocol started at 95 °C for 10 min, and then ran 40 cycles of: 95 °C for 30 s and 60 °C for 1 min. All samples were run in duplicate.

The mRNA panel consisted of 13 markers (Table 1), including sequences for: epithelial (*KRT8*, *KRT19*, *EPCAM*, E-Cadherin) and mesenchymal/EMT (vimentin, N-Cadherin, *ZEB1*) markers to differentiate epithelial CTCs from CTCs that had undergone EMT; CSC markers (*CD24*, *CD44*, *ALDH1A1*), which were previously shown to be expressed on pancreatic tumour cells with increased metastatic potential (Ishizawa et al., 2010; Li et al., 2007; Rasheed et al., 2010); the ECM marker, *SPARC*, which was demonstrated to be highly expressed in pancreatic cancer CTCs, and when knocked down in mice, it suppressed cell migration and invasiveness Ting et al. [11]; a reference marker (*HPRT1*); and a leucocyte marker (*CD45*). Cells without detectable levels of either vimentin or *SPARC* mRNA, which were expected to be expressed in all cells, were considered to have poor quality RNA, inadequate for complete mRNA profiling.

**Table 1:** mRNA panel used to analyse cell mRNA transcripts

Gene Symbol	Gene Name	ENSEMBL Gene ID	Amplicon length	Assay number
Epithelial transcripts				
*KRT8*	Keratin 8	ENSG00000170421	164	Hs01595539_g1
*KRT19*	Keratin 19	ENSG00000171345	96	AI70M8O (Custom assay)
*EPCAM*	Epithelial cell adhesion molecule	ENSG00000119888	64	Hs00158980_m1
*CDH1*	Cadherin 1; type 1, E-cadherin	ENSG00000039068	65	Hs01013953_m1
EMT-associated transcripts				
*VIM*	Vimentin	ENSG00000026025	73	Hs00185584_m1
*CDH2*	Cadherin 2; type 1, N-cadherin	ENSG00000170558	66	Hs00983056_m1
*ZEB1*	Zinc finger E-box binding homeobox 1	ENSG00000148516	63	Hs00232783_m1
Cancer stem cell transcripts				
*CD24*	CD24 molecule	ENSG00000272398	140	Hs02379687_s1
*CD44*	CD44 molecule	ENSG00000026508	70	Hs01075861_m1
*ALDH1A1*	Aldehyde dehydrogenase 1; family member A1	ENSG00000165092	61	Hs00946916_m1
Pancreas cancer-associated transcript				
*SPARC*	Secreted protein, acidic, cysteine-rich (osteonectin)	ENSG00000113140	76	Hs00234160_m1
Reference transcript				
*HPRT1*	Hypoxanthine phosphoribosyltransferase 1	ENSG00000165704	82	Hs02800695_m1
Leucocyte transcript				
*PTPRC*	Protein tyrosine phosphatase, receptor type C (CD45)	ENSG00000081237	57	Hs04189704_m1

### Statistical analysis

The statistical analyses were performed with R version 3.3.0. All reported Cq-values were expressed as the mean ± standard deviation. The reported *p*-values were calculated with the unpaired t-test, unless otherwise stated. *p*-values less than 0.05 were considered statistically significant.

Missing data points were replaced with the highest Cq-value observed for a particular gene expression assay, plus a value of 1. This approach was designed to provide balanced weighting to negative observations, and it also took into account differences in PCR efficiency among the gene expression assays. All gene expression data were also mean-centred and z-score transformed by dividing the mean-centred expression by the standard deviation; this approach provided all measured mRNAs with equal weighting in the statistical analyses. Normalization by reference marker was not performed, due to the stochastic expression of mRNAs in single cells [14, 15].

To explore associations between cell groups, we performed unsupervised hierarchical clustering and principal component analysis (PCA). Unsupervised hierarchical clustering (*Hclust* function in heatmap.2) and heatmap visualization were performed with the *heatmap*.*2* function supplied with the Gplots package in R. The unsupervised hierarchical clustering was performed with agglomerative hierarchical clustering with average (UPGMA) linkage and a distance metric equal to 1 minus the Pearson correlation. The PCA was performed with the *princomp* function in R. Figures from the PCA were constructed with the first three components, because components 1 and 2 only explained 63% of the variance.

Correlation matrix plots of correlations between the different mRNAs measured were constructed with the *corrplot* function supplied with the Corrplot package in R; it used the *cor* function to compute correlations. The correlation matrix was computed separately for CTCs, epithelial pancreatic cancer cell lines, ASPC-1 and PANC1, and the mesenchymal cell line SDM103T2, with Spearman rank correlations. Associated *p*-values were computed with the *cor*.*mtest* function in R. The Bonferroni correction of *p*-values was performed to adjust for multiple testing in the rank correlation matrix.

## Results

### Isolation and characterization of pancreatic CTCs

CTCs were detected in 33/56 (59%) peripheral blood samples from 21 patients treated for locally advanced or metastatic pancreatic cancer. Based on immunofluorescence staining, we selected 48 morphologically intact CTCs and 3 morphologically intact CTC clusters (containing ≤3 CTCs) by micromanipulation. Of these, 30/51 (59%) had sufficient RNA quality for mRNA analysis; the remaining 21/51 (41%) cells and CTC clusters had degraded RNA, despite appearing morphologically intact. In total, 18/30 (60%) cells were identified as CTCs, based on expression of epithelial and/or mesenchymal markers. For comparison, we also isolated 12 leucocytes from a healthy volunteer, and 16 single cells from each of the cell lines, PANC1, ASPC-1, and SDM103T2—all of which had been spiked into healthy blood and subjected to CTC enrichment prior to isolation.

The isolated single cells were subjected to mRNA profiling with a multi-marker mRNA panel (Table 1) designed to capture inherent heterogeneity in pancreatic CTCs. The panel consisted of sequences that identified specific epithelial markers (*KRT8*, *KRT19*, *EPCAM*, E-Cadherin); mesenchymal/EMT markers (Vimentin, N-Cadherin, *ZEB1*); CSC markers (*CD24*, *CD44*, and *ALDH1A1*); an ECM marker (*SPARC*), a reference marker (*HPRT1*), and a leucocyte marker (*CD45*).

#### Cluster analysis defines pancreatic CTCs in epithelial-like and mesenchymal-like subgroups

**Fig. 2:**
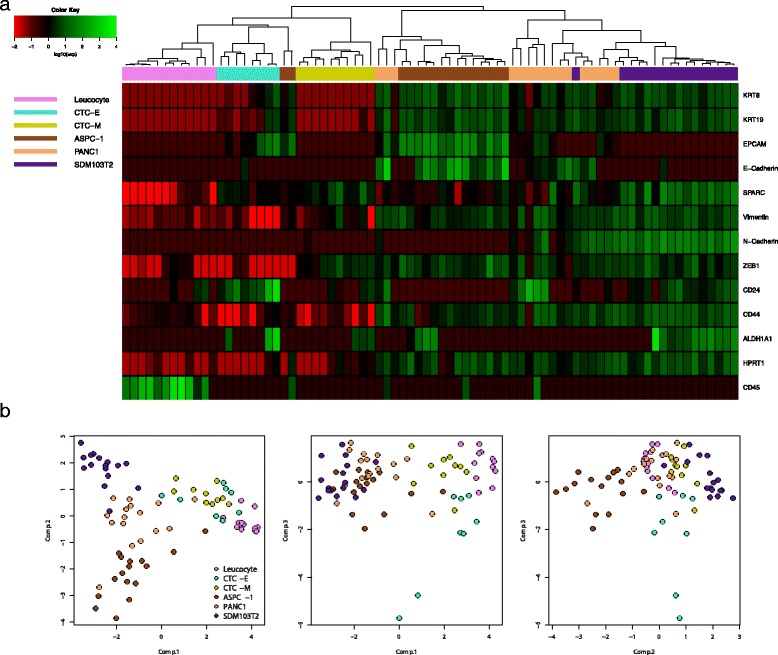
Single cell mRNA analysis of pancreatic CTCs. **a** Unsupervised hierarchical cluster analysis and associated heat map of single CTCs (*turquoise, yellow*), leucocytes (*violet*), ASPC-1 cells (*brown*), PANC1 cells (*light brown*), and SDM103T2 cells (*blue*). Data are mean-centred and z-score adjusted. *Green* and *red* colours in the heat map represent high and low expression levels, respectively, relative to the mean expression of all analysed cells. **b** Principal component analysis of the single cell data. Each point represents a single cell in the analysis

The mRNA data (Additional file 1) from all single cells were z-score adjusted and analysed with unsupervised hierarchical clustering to visualize similarities and dissimilarities (Fig. 2a). Unlike the cell-line cells, which expressed most markers in the mRNA panel at high levels, each of the CTCs expressed few markers, and generally, at lower levels than observed with cell-line cells. Based on mRNA expression patterns, the CTCs could be divided into two groups: one epithelial-like CTC cluster (CTC-E) and one mesenchymal-like CTC cluster (CTC-M). These clusters were distinct from leucocytes and cancer cell-line cells, though more closely related to the former than to the latter. From two of the patient samples, we isolated more than one CTC with sufficient mRNA quality. Two CTCs were isolated from sample PC22B3, one in each CTC group. Five CTCs were isolated from sample PC35B1, all in the CTC-E group.

The leucocytes analysed formed a separate cluster, and most of the isolated cell-line cells analysed formed separate clusters. A few cells from each cancer cell line were markedly different from all the other cell-line cells (Fig. 2a); thus, heterogeneity among single cells was observed even among apparently homogenous cancer cell-line cells. A PCA of the expression data confirmed the findings from the hierarchical clustering analysis (Fig. 2b); leucocytes, cancer cell-line cells, and the CTC subgroups formed separate clusters.

#### Expression of epithelial, mesenchymal, and CSC markers in pancreatic CTCs

Further characterization of the CTC subgroups revealed that cells in the CTC-E subgroup expressed the epithelial markers, *KRT8*, *KRT19*, and *EPCAM*, and they showed elevated expression of the CSC marker, *CD24*. These cells also lacked, or showed lower expression, of the mesenchymal markers, vimentin, *ZEB1*, and N-Cadherin. In contrast, cells in the CTC-M subgroup expressed the mesenchymal marker, *ZEB1*, showed elevated expression of vimentin compared to the cells in the CTC-E subgroup, and lacked expression of epithelial markers. Several CTCs co-expressed epithelial and mesenchymal markers; most co-expressed epithelial markers and vimentin, but a few CTCs co-expressed epithelial markers and the mesenchymal marker, *ZEB1*. These cells with an intermediate phenotype did not form a separate cluster in the hierarchical clustering analysis, but were identified in either the CTC-E or CTC-M cluster, according to their levels of mesenchymal marker expression. The CSC markers, *CD24*, *CD44*, and *ALDH1A1* were expressed in cells found in both the CTC-E and the CTC-M subgroups, and each subgroup contained cells that co-expressed two or more CSC markers. Both *CD24* and *ALDH1A1* expression levels were elevated in CTCs compared to leucocytes and pancreatic cancer cell-line cells. In contrast, *CD44* expression was similar in CTCs and leucocytes, but lower in CTCs than in cell-line cells. *CD24* expression was detected in all profiled cells in the CTC-E subgroup, and expression was elevated compared to *CD24* expression in the CTC-M subgroup (*p* < 0.001; Mann-Whitney U). Expression of the characteristic “cadherin switch” [16] proteins, E-Cadherin and N-Cadherin, was undetectable in most CTCs isolated: each of these markers was detected in only one CTC. All CTCs lacked expression of the leucocyte marker, *CD45*, but it was detected in all but one leucocyte.

**Fig. 3:**
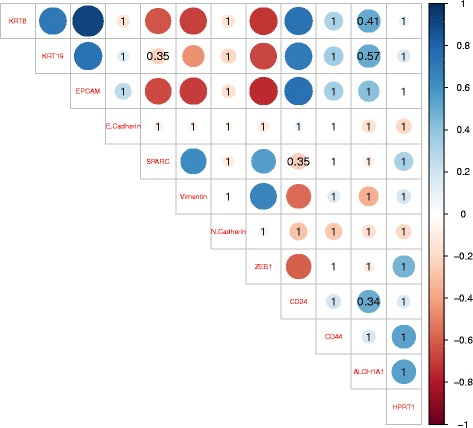
Correlation plots of mRNA expression levels in single pancreatic CTCs. Matrix shows pairwise Spearman rank correlations between expression levels of the indicated mRNAs in pancreatic CTCs. Blue and red colours represent positive and negative correlations, respectively, according to scale bar (*right*). The circle size represents the magnitude of the correlation. The values in the matrix represent *p*-values that did not reach significance. All *p*-values were corrected for multiple testing with the Bonferroni correction

A correlation analysis of the CTC mRNA levels (Fig. 3) showed high internal correlations between individual epithelial markers and between individual mesenchymal markers. Negative correlations were demonstrated between these groups, which indicated that increased expression of mesenchymal markers was associated with downregulation of epithelial markers. This finding supported the hypothesis that those CTCs were undergoing EMT. Interestingly, CSC markers were only correlated with the epithelial markers.

### High *SPARC* expression was found in pancreatic CTCs and correlated with EMT markers

The ECM marker, *SPARC*, was analysed because previous studies showed that it was elevated in pancreatic CTCs. The expression of *SPARC* was high in all isolated CTCs and cancer cell-line cells analysed, and it was nearly absent in leucocytes. On average, the expression of *SPARC* in CTCs was higher than in the pancreatic cancer cell lines, PANC1 (*p* = 0.053) and ASPC-1 (*p* = 0.004), even though the general distributions of most measured mRNAs were much lower in the CTCs than in the cell-line cells. Furthermore, we noted a trend towards higher *SPARC* expression in the CTC-M subgroup than in the CTC-E subgroup (*p* = 0.101; Mann-Whitney U). Correlation analysis of CTC mRNA levels (Fig. 3) revealed that *SPARC* expression was moderately correlated with the EMT markers, vimentin (Spearman correlation = 0.62, *p* = 0.0003) and *ZEB1* (Spearman correlation = 0.55, *p* = 0.01), and moderately negatively correlated with the epithelial markers, *KRT8* (Spearman correlation = −0.63, *p* = 0.014) and *EPCAM* (Spearman correlation = −0.65, *p* = 0.004). These correlations were not observed in the pancreatic cancer cell-line cells (Additional file 2) or in the mesenchymal cancer cell-line cells (Additional file 3).

### Clinical relevance of CTC-E and CTC-M detection

CTCs with sufficient mRNA quality were isolated from 13 samples obtained from 8 patients. The CTC-E phenotype was present in four samples obtained from four patients. For three of these patients follow-up data was available. These three patients had a median survival, from the time of inclusion, of 10.6 months (95% CI: 0–22.2). In contrast, the CTC-M phenotype was encountered more frequently; it was present in 10 samples obtained from seven patients. In four of these patients, the CTC-E phenotype was not present at any time point during follow-up. The median survival of these four patients, from the time of inclusion, was 18.5 months (95% CI: 14.8–22.2). The difference in survival between patients where the CTC-E phenotype was present during follow-up and patients where only the CTC-M phenotype was present was borderline significant (log-rank test: *p* = 0.093). Interestingly, both patients that provided more than one isolated CTC in a single sample died within 3 months after CTC detection. In total, 3 of 4 patients with CTC-E positive samples progressed at the same time or shortly after the positive sample were identified, and they died within 3 months. In contrast, although some patients progressed after a CTC-M positive sample was identified, only 1 of 5 patients died shortly after providing a sample positive only for CTC-M. This patient was hospitalized with sepsis and died from toxicity due to the chemotherapy.

## Discussion

With our previously established CTC enrichment strategy, MINDEC [12], and single-cell mRNA profiling, we isolated and garnered gene expression data from single human pancreatic CTCs. In total, we isolated and profiled 18 CTCs from 13 patient samples. Although the mRNA panel was small, with only 13 markers, the CTC clustering analysis revealed two distinct subpopulations, CTC-E and CTC-M. These subpopulations were distinct from both leucocytes and cell-line cells. Ting et al. previously stratified mouse pancreatic CTCs into classic (epithelial), proliferative, and platelet-associated subgroups, based on mRNA expression [11]. However, to our knowledge, this study was the first to describe a subgroup of human pancreatic CTCs enriched for mesenchymal markers, based on mRNA expression. However, previous studies have described CTCs with mesenchymal and intermediate epithelial-mesenchymal phenotypes, based on protein expression [17, 18].

A large number of the CTCs isolated (41%) had low quality RNA, inadequate for mRNA profiling. We suspected that these cells might have lost viability during the enrichment process. However, mRNA profiles were obtained for all isolated cell-line cells after the spiking experiments. Thus, we suspected that it was more likely that CTCs with degraded RNA lost viability in the bloodstream, prior to sampling and enrichment. Similar numbers of CTCs with insufficient mRNA quality were also reported by other groups that employed other enrichment methods [11, 19].

Both *CD24* and *CD44* were frequently expressed in CTCs, but CTCs did not express higher *CD44* levels than leucocytes. *ALDH1A1* was expressed in several CTCs, and several cells also co-expressed two or three CSC markers. Sorted pancreatic tumour cells ALDH-positive, dual CD24- and CD44-positive, and triple CD24-, CD44-, and ALDH-positive were previously demonstrated to be highly tumourigenic compared to unsorted tumour cells. As few as 100 of the sorted cells were necessary to produce tumours after xenotransplantation in immuno-compromised mice [20–22]. Although we measured mRNA levels, which do not necessarily translate to protein levels, the prevalence of CSC markers in CTCs suggested that these cells may have high metastatic potential. Accordingly, the high prevalence of CSC markers in pancreatic CTCs might explain the high metastatic frequency of pancreatic tumours, despite the low number of CTCs reported [17, 23, 24].

*SPARC* was highly expressed in all isolated CTCs compared to cell-line cells and leucocytes, particularly in the CTC-M group. This finding suggested that *SPARC* upregulation might be related to the ability of CTCs to spread and invade distant sites. *SPARC* mRNA levels were previously demonstrated to be highly elevated in both chronic pancreatitis (16-fold increase) and pancreatic cancers (31-fold increase), compared to normal pancreatic tissue [25]. Elevated SPARC protein levels were also demonstrated to promote invasiveness of pancreatic tumour cells. In contrast to our findings, Ting et al., in a mouse model, found that *SPARC* expression was highest in epithelial–like CTCs [11]. They also demonstrated that *SPARC* was highly expressed in human pancreatic CTCs, and they provided evidence of the invasiveness and metastatic potential of SPARC-expressing tumour cells. In contrast, our results suggested that *SPARC* expression was associated with mesenchymal markers and CTCs undergoing EMT, because *SPARC* expression was positively correlated with vimentin and *ZEB1* and negatively correlated with *KRT8* and *EPCAM*. Consistent with our results, evidence from melanoma and breast carcinoma studies pointed to SPARC as an inducer of EMT [26, 27].

It should be taken into consideration that our single-cell mRNA analyses might have been affected by burst transcription in single cells. This process causes the levels of specific mRNAs to fluctuate over time [14, 15], and it is the primary cause of differences in mRNA expression levels between apparently identical cells. The nature of burst transcription precludes the normalization of quantitative RT-PCR data from single cells against a reference transcript [28]. Nevertheless, it was demonstrated that the magnitude of the noise caused by burst transcription was smaller than the variation caused by gene regulation [29].

The number of CTCs we isolated from each patient sample in this study was consistent with previous reports on pancreatic CTCs [17, 23, 24]. The low number of detectable CTCs in pancreatic cancer is most likely due to their uptake by the liver, which occurs when the venous drainage from the gastrointestinal tract passes through the liver, which is prone to metastatic spread in pancreatic cancer. Catenacci et al. reported that the number of CTCs in patients with pancreaticobiliary cancers was more than 100-fold higher in portal vein blood compared to peripheral blood. That finding suggested that enumerating CTCs in peripheral blood from patients with pancreatic tumours might be difficult compared to other solid cancers [24]. Ideally, portal vein blood would be the best source of blood for CTC analysis in patients with pancreatic cancer. However, acquisition of portal vein blood is an invasive procedure that would be hard to recommend for a patient group with high morbidity.

Several previous investigations of pancreatic CTCs have failed to support the clinical utility of CTC analysis in pancreatic cancer. However, the consensus opinion was that CTC analysis in pancreatic cancer is clinically relevant (reviewed in [6, 7]). Although some previous studies were limited by small patient cohorts, it is apparent that, in most studies, CTCs were detected with only a single or few markers, which either targeted epithelial- or cancer-specific transcripts or proteins. Thus, those results did not elucidate the complexity of the identified CTCs. In this study, we showed that CSC and mesenchymal transcripts were detected in the majority of isolated CTCs. Similar to our findings, a previous study described a subgroup of CTCs with a mesenchymal profile and CTCs that expressed CSC transcripts in patients with breast cancer [30]. In a recent study by Poruk et al., the expression of CSC markers on the surface of pancreatic epithelial CTCs was associated with shorter disease-free and overall survival [31]. Evidence from research on colorectal and breast cancer has also suggested that the identification of mesenchymal CTCs and transient CTCs undergoing EMT may provide additional prognostic information, compared to the information provided by epithelial CTCs alone [32–34]. The prognostic value of mesenchymal CTCs in pancreatic cancer is not clear; however, in a study by Poruk et al., the detection of vimentin on CK-positive CTCs was associated with disease recurrence [18]. Our data demonstrated that the CTC-E group expressed higher levels of stem cell markers than the CTC-M group. We also found indications that CTC-Es may predict survival better than CTC-Ms. However, due to the small patient numbers in that analysis, we urge careful interpretation; these results should be validated in a larger patient cohort.

Recent evidence has also suggested that CTC clusters might be a better prognostic marker than CTCs. Chang et al. detected CTC clusters in patients with pancreatic cancer and found them to be independent predictors of progression-free and overall survival [35]. We isolated three CTC clusters from three different patient samples, but only one cluster had sufficient mRNA quality for analysis. These low numbers prevented us from drawing any conclusions about whether CTC clusters might yield additional clinical information.

The major limitation of our study was the small mRNA panel used. This panel was designed to differentiate between epithelial and mesenchymal CTCs, and to assess the presence of potential tumourigenic CTCs, based on CSC marker expression. In hindsight, the mRNA panel could well have included more markers to provide a better characterization of CTCs. In a recent study on mRNA heterogeneity in single CTCs, Gorges et al. included markers associated with resistance to cancer therapy and tumour progression in their mRNA panel. Those markers were readily detected in breast and prostate cancer CTCs [30]. Inclusion of such markers in our mRNA panel might have provided a means to identify the CTC groups responsible for metastasis. In addition, such markers might have provided information on tumour progression and the effect of therapy. However, future single-cell analyses of pancreatic CTCs might be best served by performing RNA sequencing, which could avoid dependence on small, selective mRNA panels [11]. Other limitations to our study included the low numbers of analysed CTCs and the lack of patients with localized disease in our patient cohort. Moreover, it would have been interesting to analyse CTCs in early-stage cancer samples, for instance after surgery, to determine whether specific CTC subpopulations also have prognostic value for these patients.

## Conclusions

In conclusion, our analysis of single pancreatic CTCs identified distinct epithelial-like and mesenchymal-like subpopulations and revealed elevated expression of CSC markers and the EMT-associated marker, *SPARC*. These transcripts might be relevant in the dissemination of CTCs to distant organ sites. In addition, our preliminary results suggested that epithelial-like CTCs may be a better predictor of survival than mesenchymal-like CTCs. That finding warrants future investigations in larger studies.

## Additional files

Additional file 1: Single-cell Cq values for all cells analysed. (DOCX 36 kb)

Additional file 2: Correlation plots of mRNA expression levels in single pancreatic cancer cell-line cells. (DOCX 120 kb)

Additional file 3: Correlation plots of mRNA expression levels in single mesenchymal cancer cell-line cells. (DOCX 106 kb)

## Abbreviations

CSC: Cancer stem cells
CTC: Circulating tumour cell
CTC-E: Epithelial-like CTC
CTC-M: Mesenchymal-like CTC
ECM: Extracellular matrix
EMT: Epithelial-mesenchymal transition
