# Supplementary material for: Single-cell mRNA profiling reveals transcriptional heterogeneity among pancreatic circulating tumour cells
Source: BMC Cancer. 2017 May 31;17:390. doi: 10.1186/s12885-017-3385-3 (PMC5452374; doi:10.1186/s12885-017-3385-3)
Supplement: Supplementary file 1 — Single-cell Cq values for all cells analysed. (DOCX 36 kb) [file 12885_2017_3385_MOESM1_ESM.docx]

| **Additional file 1: Single-cell Cq for all cells analysed** | | | | | | | | | | | | | |
| --- | --- | --- | --- | --- | --- | --- | --- | --- | --- | --- | --- | --- | --- |
| **Cell** | **KRT8** | **KRT19** | **EPCAM** | **E-Cadherin** | **SPARC** | **Vimentin** | **N-Cadherin** | **ZEB1** | **CD24** | **CD44** | **ALDH1A1** | **HPRT1** | **CD45** |
| PC22B3_Cell_1 | N/A | 34,47 | N/A | N/A | 27,39 | 29,25 | N/A | N/A | 35,58 | N/A | N/A | N/A | N/A |
| PC22B3_Cell_3 | N/A | N/A | N/A | N/A | 28,23 | 27,09 | N/A | 31,45 | N/A | N/A | 33,70 | 35,57 | N/A |
| PC21B5_Cell_3 | N/A | N/A | N/A | N/A | 28,00 | 27,50 | N/A | 34,59 | 31,36 | N/A | N/A | N/A | N/A |
| PC21B6_Cell_4 | N/A | N/A | N/A | N/A | 27,55 | 28,12 | N/A | 33,79 | N/A | 33,10 | N/A | N/A | N/A |
| PC18B6_Cell_5 | N/A | N/A | N/A | N/A | 26,50 | 26,36 | N/A | 31,58 | 35,59 | 31,35 | N/A | 35,59 | N/A |
| PC25B3_Cell_4 | N/A | 33,32 | N/A | N/A | 25,19 | 25,38 | N/A | 31,49 | 35,14 | 32,60 | 33,92 | 33,95 | N/A |
| PC18B9_Cell_3 | N/A | N/A | N/A | N/A | 28,30 | 28,62 | 34,32 | 33,08 | N/A | N/A | N/A | N/A | N/A |
| PC25B5_Cell_2 | N/A | N/A | N/A | N/A | 28,80 | 30,44 | N/A | 33,41 | N/A | 35,16 | N/A | N/A | N/A |
| PC14B17_Cell_2 | N/A | N/A | N/A | N/A | 28,18 | 27,70 | N/A | 32,06 | N/A | 33,77 | N/A | 35,13 | N/A |
| PC25B7_Cell_1 | N/A | N/A | N/A | N/A | 25,26 | 23,49 | N/A | 30,22 | 37,64 | 31,01 | N/A | 33,12 | N/A |
| PC16B16_Cell_1 | N/A | N/A | N/A | N/A | 28,73 | 27,01 | N/A | 31,69 | N/A | 33,31 | N/A | N/A | N/A |
| PC25B9_Cell_2 | N/A | 34,73 | 33,05 | 36,09 | 28,28 | 29,87 | N/A | 34,81 | 34,83 | 33,65 | N/A | N/A | N/A |
| PC35B1_Cell_3 | 30,42 | 28,73 | 27,32 | N/A | 28,62 | N/A | N/A | N/A | 28,30 | 30,18 | 30,21 | 35,12 | N/A |
| PC35B1_Cell_4 | 33,03 | 34,48 | 28,93 | N/A | 28,94 | N/A | N/A | N/A | 33,33 | 32,68 | N/A | N/A | N/A |
| PC35B1_Cell_cluster_1 | 37,21 | 34,67 | 33,08 | N/A | 28,18 | 30,59 | N/A | N/A | 33,41 | N/A | 33,12 | N/A | N/A |
| PC35B1_Cell_5 | 34,48 | 35,58 | 32,46 | N/A | 30,76 | 33,17 | N/A | N/A | 33,70 | N/A | N/A | N/A | N/A |
| PC35B1_Cell_6 | 27,20 | 28,38 | 25,95 | N/A | 29,00 | 32,75 | N/A | N/A | 25,41 | 29,20 | 28,40 | 33,79 | N/A |
| PC39B3_Cell_1 | N/A | N/A | N/A | N/A | 27,73 | N/A | N/A | 31,35 | 34,30 | N/A | 33,58 | 34,62 | N/A |
| WBC CD45 pos 1 | N/A | N/A | N/A | N/A | 33,51 | 29,44 | N/A | 33,00 | 36,36 | 31,09 | N/A | N/A | 33,48 |
| WBC CD45 pos 2 | N/A | N/A | N/A | N/A | N/A | 30,44 | N/A | 36,74 | N/A | 31,26 | N/A | N/A | 34,30 |
| WBC CD45 pos 3 | N/A | N/A | N/A | N/A | N/A | 29,57 | N/A | 34,47 | N/A | 30,16 | N/A | N/A | 35,07 |
| WBC CD45 pos 4 | N/A | N/A | N/A | N/A | N/A | 31,06 | N/A | N/A | N/A | 33,23 | N/A | N/A | 35,59 |
| WBC CD45 pos 5 | N/A | N/A | N/A | N/A | N/A | 30,04 | N/A | N/A | N/A | 33,13 | N/A | 35,66 | N/A |
| WBC CD45 pos 6 | N/A | N/A | N/A | N/A | N/A | 30,52 | N/A | N/A | N/A | 31,95 | N/A | N/A | 35,27 |
| WBC CD45 pos 7 | N/A | N/A | N/A | N/A | 31,14 | 29,45 | N/A | 33,68 | N/A | 30,88 | N/A | N/A | 33,29 |
| WBC CD45 pos 8 | N/A | N/A | N/A | N/A | 30,19 | 27,95 | N/A | 33,76 | N/A | 29,98 | N/A | 35,71 | 34,31 |
| WBC CD45 pos 9 | N/A | N/A | N/A | N/A | N/A | 30,97 | N/A | N/A | N/A | 31,09 | N/A | 37,28 | 34,12 |
| WBC CD45 dim 1 | N/A | N/A | N/A | N/A | N/A | 28,61 | N/A | N/A | N/A | 30,23 | N/A | 35,77 | 35,97 |
| WBC CD45 dim 2 | N/A | N/A | N/A | N/A | 31,32 | 29,67 | N/A | N/A | 34,12 | N/A | N/A | N/A | 35,25 |
| WBC CD45 dim 3 | N/A | N/A | N/A | N/A | 29,94 | 31,56 | N/A | N/A | 34,94 | 32,07 | N/A | N/A | 36,77 |
| PANC1 1 | 38,29 | 33,35 | 33,50 | 37,31 | 31,66 | 27,69 | 34,37 | 34,41 | 27,94 | 32,11 | N/A | 33,77 | N/A |
| PANC1 2 | 26,55 | 25,45 | 27,15 | 30,05 | 28,52 | 23,63 | N/A | 30,95 | 31,37 | 23,65 | N/A | 31,00 | 36,69 |
| PANC1 3 | 35,30 | 33,76 | 31,57 | N/A | 30,10 | 23,35 | N/A | 32,70 | N/A | 28,69 | N/A | 33,09 | N/A |
| PANC1 4 | 28,55 | 28,92 | 30,11 | 35,98 | 31,46 | 21,69 | 28,86 | 30,47 | 30,24 | 25,55 | N/A | 30,89 | N/A |
| PANC1 5 | 34,74 | 30,07 | 30,92 | N/A | 29,51 | 24,10 | 30,21 | 31,50 | N/A | 27,72 | N/A | 31,36 | N/A |
| PANC1 6 | 27,92 | 27,94 | N/A | 33,90 | 28,29 | 23,72 | 29,40 | 31,20 | N/A | 25,50 | N/A | 32,09 | N/A |
| PANC1 7 | 28,39 | 27,82 | N/A | 33,32 | 29,49 | 25,26 | 32,51 | 34,82 | N/A | 27,44 | N/A | 33,22 | N/A |
| PANC1 8 | 32,31 | 31,69 | 34,07 | N/A | 29,28 | 27,06 | 33,11 | 32,46 | 34,99 | 28,50 | N/A | 31,67 | N/A |
| PANC1 9 | 28,00 | 29,60 | 33,14 | 33,86 | 31,72 | 23,48 | 32,75 | 32,42 | 35,47 | 26,60 | N/A | 32,26 | N/A |
| PANC1 10 | 29,53 | 28,80 | 34,46 | N/A | 28,28 | 22,20 | 29,45 | 31,14 | N/A | 25,20 | N/A | 30,06 | N/A |
| PANC1 11 | 29,17 | 28,57 | N/A | 35,96 | 26,98 | 24,14 | 30,67 | 31,99 | N/A | 27,36 | N/A | 32,15 | N/A |
| PANC1 12 | 32,22 | 30,30 | N/A | 34,63 | 28,63 | 26,14 | 33,86 | 33,28 | N/A | 28,33 | N/A | 33,45 | N/A |
| PANC1 13 | 32,61 | 28,57 | 30,14 | 36,36 | 29,34 | 21,74 | 31,39 | 30,89 | 29,69 | 25,05 | N/A | 30,89 | 35,49 |
| PANC1 14 | 28,70 | 28,15 | 33,68 | 36,43 | 24,49 | 23,68 | N/A | 31,60 | N/A | 25,51 | N/A | 32,27 | N/A |
| PANC1 15 | 33,78 | 29,04 | 32,94 | N/A | 29,14 | 24,11 | 30,21 | 30,84 | 35,96 | 26,15 | N/A | 30,77 | N/A |
| PANC1 16 | 30,82 | 29,34 | 33,72 | N/A | 28,12 | 22,73 | N/A | 29,99 | 32,29 | 26,18 | N/A | 31,57 | N/A |
| ASPC-1 1 | 27,99 | 27,10 | 26,22 | N/A | 29,17 | 24,26 | N/A | 30,61 | N/A | 26,57 | 35,62 | 29,47 | N/A |
| ASPC-1 2 | 26,27 | 26,71 | 25,27 | 32,43 | 29,95 | 26,15 | N/A | 32,69 | N/A | 31,43 | 31,29 | 31,80 | N/A |
| ASPC-1 3 | 29,53 | 28,53 | 27,24 | N/A | 28,96 | 26,70 | N/A | N/A | N/A | 30,60 | N/A | 32,49 | 36,23 |
| ASPC-1 4 | 28,55 | 27,43 | 29,82 | 35,35 | 29,63 | 24,10 | N/A | 31,50 | N/A | 27,45 | N/A | 31,58 | N/A |
| ASPC-1 5 | 27,63 | 28,23 | 28,06 | 34,84 | 28,21 | 24,80 | N/A | 30,66 | N/A | 27,96 | N/A | 32,79 | N/A |
| ASPC-1 6 | 27,75 | 28,74 | 26,34 | 34,80 | 30,24 | 27,08 | N/A | 31,23 | N/A | 32,75 | 32,65 | 33,28 | N/A |
| ASPC-1 7 | 34,10 | 31,50 | 30,83 | N/A | 29,74 | 27,97 | N/A | N/A | N/A | 31,35 | N/A | N/A | N/A |
| ASPC-1 8 | 26,13 | 27,67 | 25,32 | 33,20 | 30,29 | 24,25 | N/A | 31,37 | N/A | 28,80 | 31,60 | 30,69 | N/A |
| ASPC-1 9 | 26,01 | 27,64 | 25,74 | 35,70 | 28,71 | 24,50 | N/A | 31,98 | N/A | 28,14 | N/A | 30,29 | N/A |
| ASPC-1 10 | 24,86 | 25,72 | 25,09 | 31,30 | 32,92 | 25,20 | N/A | 31,38 | N/A | 28,11 | N/A | 30,12 | N/A |
| ASPC-1 11 | 28,13 | 30,15 | 28,08 | 31,97 | 28,79 | 25,42 | N/A | 31,31 | N/A | 26,82 | N/A | 34,54 | N/A |
| ASPC-1 12 | 24,63 | 25,79 | 24,36 | 30,29 | 27,17 | 23,89 | N/A | 30,16 | N/A | 27,46 | N/A | 30,42 | N/A |
| ASPC-1 13 | 27,97 | 27,24 | 25,09 | 32,32 | 29,17 | 24,84 | N/A | 32,24 | N/A | 28,42 | N/A | 30,83 | 36,24 |
| ASPC-1 14 | 30,87 | 29,38 | 28,08 | 34,91 | 27,84 | 23,93 | N/A | 30,40 | N/A | 28,24 | N/A | 35,64 | N/A |
| ASPC-1 15 | 28,22 | 27,25 | 26,03 | N/A | 29,31 | 22,28 | N/A | 29,39 | N/A | 27,39 | N/A | 31,69 | N/A |
| ASPC-1 16 | 26,57 | 26,12 | 25,30 | 33,23 | 30,28 | 22,71 | N/A | 27,79 | N/A | 26,26 | N/A | 29,93 | N/A |
| SDM103T2 1 | 27,80 | 26,59 | N/A | 34,56 | 24,63 | 24,21 | 31,53 | 31,75 | 36,68 | 26,48 | N/A | 31,42 | N/A |
| SDM103T2 2 | 27,78 | 27,85 | N/A | N/A | 25,80 | 24,51 | 30,03 | 32,75 | 34,05 | 26,57 | 32,43 | 32,57 | N/A |
| SDM103T2 3 | 26,27 | 26,61 | N/A | N/A | 23,28 | 22,45 | 28,15 | 29,21 | 34,81 | 25,58 | N/A | 28,96 | N/A |
| SDM103T2 4 | 27,98 | 26,96 | N/A | N/A | 24,47 | 22,46 | 28,64 | 29,14 | 35,04 | 25,22 | 31,71 | 29,07 | N/A |
| SDM103T2 5 | 27,68 | 27,59 | N/A | 35,99 | 23,40 | 23,30 | 27,61 | 32,28 | 34,81 | 25,59 | 32,43 | 30,28 | N/A |
| SDM103T2 6 | 29,96 | 27,00 | N/A | N/A | 25,00 | 24,18 | 28,95 | 31,49 | 33,44 | 25,40 | 34,18 | 31,23 | N/A |
| SDM103T2 7 | 31,09 | 29,30 | N/A | N/A | 25,86 | 24,77 | 29,70 | 32,51 | 35,81 | 27,53 | 31,90 | 32,10 | N/A |
| SDM103T2 8 | 27,23 | 25,57 | N/A | N/A | 24,92 | 24,03 | 28,54 | 32,41 | 32,09 | 25,74 | 33,58 | 30,67 | N/A |
| SDM103T2 9 | 31,04 | 29,76 | N/A | N/A | 27,86 | 25,03 | 30,52 | 31,38 | 35,90 | 27,85 | N/A | 32,31 | N/A |
| SDM103T2 10 | 27,62 | 30,28 | N/A | N/A | 28,82 | 22,19 | 29,50 | 30,50 | N/A | 24,70 | 27,67 | 31,96 | N/A |
| SDM103T2 11 | 27,06 | 27,97 | N/A | N/A | 25,65 | 22,97 | 28,82 | 30,85 | N/A | 25,79 | N/A | 30,23 | N/A |
| SDM103T2 12 | 27,80 | 27,40 | N/A | N/A | 24,61 | 21,71 | 29,15 | 30,60 | 35,67 | 26,59 | N/A | 32,96 | N/A |
| SDM103T2 13 | 26,90 | 26,53 | N/A | N/A | 23,42 | 22,83 | 27,44 | 30,99 | 32,58 | 25,35 | 31,28 | 29,10 | N/A |
| SDM103T2 14 | 27,66 | 27,33 | N/A | N/A | 24,39 | 23,60 | 28,29 | 30,83 | 34,94 | 26,07 | 31,29 | 29,90 | N/A |
| SDM103T2 15 | 28,92 | 28,59 | N/A | N/A | 26,33 | 22,64 | 28,43 | 30,66 | 34,95 | 25,88 | 33,50 | 31,11 | N/A |
| SDM103T2 16 | 27,21 | 26,73 | N/A | N/A | 24,78 | 22,83 | 28,35 | 31,30 | 33,24 | 25,10 | 32,34 | 30,04 | N/A |
