# Supplementary material for: Single-cell mRNA profiling reveals transcriptional heterogeneity among pancreatic circulating tumour cells
Source: BMC Cancer. 2017 May 31;17:390. doi: 10.1186/s12885-017-3385-3 (PMC5452374; doi:10.1186/s12885-017-3385-3)
Supplement: Supplementary file 3 — Correlation plots of mRNA expression levels in single mesenchymal cancer cell-line cells. (DOCX 106 kb) [file 12885_2017_3385_MOESM3_ESM.docx]

**Additional file 3: Correlation plots of mRNA expression levels in single mesenchymal cancer cell-line cells.** Matrix shows pairwise Spearman rank correlations between expression levels of the indicated mRNAs in mesenchymal cancer cell-line cells (SDM103T2). Blue and red colours represent positive and negative correlations, respectively, according to scale bar (*right*). The circle size represents the magnitude of the correlation. The values in the matrix represent p–values that did not reach significance. All p–values were corrected for multiple testing with the Bonferroni correction.
